# Precise Time Synchronization in Packet Networks Using Deep Learning for Future Intelligent Transportation

**DOI:** 10.3390/s26092758

**Published:** 2026-04-29

**Authors:** Hui Deng, Haotian Li, Zesong Tian, Jun Tian, Wen Du

**Affiliations:** 1 College of Intelligence and Computing, Tianjin University, Tianjin 300072, China; fjdenghui1@chinatelecom.cn; 2 China Telecom Digital City Technology Co., Ltd., Xiong án New Area 071700, China; 3 Beijing University of Posts and Telecommunications, Beijing 100876, China; 4 Xiongan National Center of Technology Innovation Co., Ltd., Xiong án New Area 071700, China; tianjun2035@126.com (J.T.); 18632066875@163.com (W.D.)

**Keywords:** precise time synchronization, deep reinforcement learning, intelligent transportation systems, proximal policy optimization, clock drift

## Abstract

Precise time synchronization is foundational for future intelligent transportation systems (ITS), where safety-critical functions like cooperative Vehicle-to-Everything (V2X) communication and multi-sensor fusion demand a leap from sub-microsecond- to nanosecond-level precision. Standard protocols like the Precision Time Protocol (PTP) are limited by inherent errors (e.g., timestamping inaccuracies and clock drift) that are typically only solvable with expensive hardware upgrades. This paper proposes a cost-effective, software-based solution. We introduce a novel method that leverages deep reinforcement learning (DRL) to actively predict and compensate for these synchronization errors in real time. An experimental environment is constructed to rigorously evaluate the performance of the proposed method. The results demonstrate that our approach achieves a significant leap in synchronization accuracy, showcasing its potential to meet the stringent timing demands of future intelligent transportation.

## 1. Introduction

Sub-microsecond time synchronization in packet networks has established a critical foundation for deterministic communication [1]. However, no domain is pushing the boundaries of this precision more aggressively than future intelligent transportation systems (ITS), where precise timing is interchangeable with safety and efficiency. Basic applications, such as on-board sensor fusion (LiDAR, cameras, and radar) in autonomous vehicles, already require sub-microsecond accuracy to align sensor data and avoid positional discrepancies at high speeds [2].

The true challenge, however, emerges with advanced Vehicle-to-Everything (V2X) communication. Applications like cooperative perception (allowing vehicles to “see” through obstacles) and high-density platooning, where event-triggered communication is employed to optimize bandwidth [3], require vehicles and infrastructure to share a unified time base with unprecedented accuracy. The most demanding applications, such as V2X-based cooperative positioning, rely on measuring the time-of-flight of wireless signals to achieve centimeter-level accuracy. Given that radio signals travel at approximately 0.3 m per nanosecond, an uncorrected time error of just 10 ns can result in a 3 m positioning error, rendering the system unsafe and ineffective [4]. This escalates the requirement from the microsecond regime squarely into the low-nanosecond (ns) regime.

This stringent nanosecond requirement is echoed by the very technologies expected to support ITS. Future 6G cellular networks, which will underpin many V2X services, envision features like Coordinated Multi-Point (CoMP) that also depend critically on nanosecond-level time alignment between nodes [5,6]. This pursuit of ultimate precision is mirrored in other domains, such as large-scale scientific research (e.g., particle accelerators), which have long required event correlation at the nanosecond scale [7].

The standard mechanism for achieving time synchronization within packet-based frameworks is the Precision Time Protocol (PTP, IEEE 1588) or its profiles like IEEE 802.1AS [8,9]. While effectively delivering baseline sub-microsecond accuracy, conventional PTP implementations face inherent challenges when pushed towards the ultra-high precision required by cutting-edge applications. Primary limitations stem from error sources intrinsic to the protocol’s operation and typical network hardware, most notably inaccuracies introduced during the timestamping of synchronization messages [10] and the continuous drift of local clocks between PTP correction intervals. Mitigating these errors sufficiently to break into the low-nanosecond range often involves deploying expensive hardware solutions, such as network interfaces with dedicated hardware timestamping capabilities and highly stable, costly oscillators (e.g., OCXOs) throughout the network [11]. This creates the following significant barrier: the need for enhanced synchronization performance often conflicts with the practical requirement for cost-effective and scalable deployment.

This paper directly addresses the critical need for achieving ultra-high timing precision in packet-based systems in a cost-effective manner, with a focus on enabling future ITS applications. The main contributions are as follows:

1. This paper provides an in-depth analysis of the primary error sources inherent in the PTP/gPTP synchronization process, identifying timestamp processing errors and clock drift within the synchronization period as key factors limiting synchronization accuracy.
2. Addressing these identified errors, an innovative high-precision time synchronization method based on deep learning is proposed. This method utilizes a deep learning model to perform real-time prediction of errors occurring during the synchronization process and applies active compensation.
3. Through the construction of a dedicated experimental environment and subsequent testing, the results demonstrate that the proposed method achieves significant performance improvements, effectively surpassing the conventional accuracy limits prevalent in traditional packet networks.

The rest of this paper is organized as follows. Section 2 reviews the related work in network time synchronization. Section 3 formulates the problem, covering the system model, problem description, and optimization target. Section 4 describes the proposed deep reinforcement learning-enhanced precise time synchronization method. Section 5 presents the experiments and results. Finally, Section 6 concludes this paper.

## 2. Related Works

Improving the performance of time synchronization through compensation has attracted the attention of many researchers, and many time synchronization compensation schemes for the PTP protocol have been proposed. For example, some studies aim to correct path asymmetry by measuring the ingress and egress delays of PTP clocks, effectively reducing the average synchronization error [12]. Other works concentrate on directly compensating for the impact of disturbances on time synchronization, achieving a synchronization accuracy better than 10 nanoseconds [13]. Furthermore, network time synchronization architectures based on Software-Defined Networking (SDN) have been introduced, utilizing a synchronization controller to manage the synchronization process and precision compensation. In devices supporting hardware timestamping, the synchronization error can be controlled between 10 and 40 nanoseconds [14]. In the context of Time-Sensitive Networking (TSN), research has also explored the use of neural networks for time synchronization compensation, achieving an overall synchronization deviation reduction to approximately 15 nanoseconds [15]. Addressing the issue of multi-domain synchronization in TSN, researchers have proposed multi-domain time synchronization models and designed software-defined time compensation methods to centrally correct time errors, demonstrating significant error reduction in various test scenarios [16]. Additionally, a cross-domain interconnection scheme for TSN based on a coordinate controller has been proposed, controlling the time synchronization error below 50 nanoseconds [17].

In recent years, reinforcement learning has been widely used in various complex control and optimization problems. Ref. [18] demonstrates an ultra-high precision time synchronization scheme by reducing link asymmetry for cloud radio over fiber network (C-RoFN) in beyond 5G, which is supported by a deep reinforcement learning (DRL)-based autonomous synchronous signal routing algorithm. The proposed scheme achieves < 100 ns synchronization accuracy by simulation. Ref. [19] proposes a novel clock servo mechanism based on reinforcement learning, and simulation results demonstrate that the RL-based clock servo can reduce synchronization error from ±10 ms to ±100 μs.

## 3. Problem Formulation

### 3.1. Frequency Skew and Drift Decomposition

**Figure 1:**
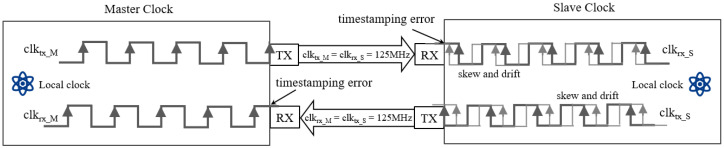
Timestamping error and clock drift in the PTP process.

As shown in Figure 1, the synchronization architecture relies on discrete crystal oscillators (typically 125 MHz). The time errors are driven by the frequency discrepancy between master and slave. We decompose the instantaneous frequency deviation $f_{dev}\left(t\right)$ into three components as follows:

(1) $$\begin{array}{c} f_{dev}\left(t\right)=\beta_{skew}+\alpha_{drift}\left(t\right)+\epsilon_{rw}\left(t\right) \end{array}$$

Here, $\beta_{skew}$ denotes static frequency skew from hardware tolerances, manifesting as a constant rate compensated by standard PTP. The term $\epsilon_{rw}\left(t\right)$ represents high-frequency random walk noise, which is typically negligible over short control intervals.

The primary challenge stems from stochastic frequency drift $\alpha_{drift}\left(t\right)$ caused by environmental factors (e.g., temperature variations). This drift is modeled as an Ornstein–Uhlenbeck (OU) process as follows:

(2) $$\begin{array}{c} d\alpha_{drift}\left(t\right)=\theta \left(\mu -\alpha_{drift}\left(t\right)\right)dt+\sigma dW_{t} \end{array}$$

where θ is the mean-reversion speed, μ is the long-term mean frequency, and σ is the volatility of the Wiener process $W_{t}$. This formulation confirms that the cumulative time error follows a non-linear stochastic trajectory.

### 3.2. Timestamping Error and Distributional Convergence Analysis

Precision is constrained by discrete signal sampling and physical noise. Timestamping triggers at the rising edge of the clock cycle $T_{clk}$. For a true packet arrival time ϕ(t) (in seconds), the idealized quantization error $n_{q}\left(t\right)$ is modeled as follows:

(3) $$\begin{array}{c} n_{q}\left(t\right)=Q\left(\phi \left(t\right)\right)=\left\lfloor \phi \left(t\right)\cdot f_{clk}+\frac{1}{2}\right\rfloor \cdot T_{clk}-\phi \left(t\right) \end{array}$$

Assuming packet arrival times are statistically independent of the clock phase, $n_{q}$ follows a uniform distribution $n_{q}∼U\left[-\frac{T_{clk}}{2},\frac{T_{clk}}{2}\right]$.

Using four timestamps ($t_{1},t_{2},t_{3},t_{4}$) from a two-way transaction, the standard PTP offset is calculated as follows:

(4) $$\begin{array}{c} \mathrm{Offset}=\frac{\left(t_{2}-t_{1}\right)-\left(t_{4}-t_{3}\right)}{2} \end{array}$$

Decomposing intervals into symmetric propagation delay *d* and quantization noise terms ($n_{ms},n_{sm}$) as follows:

(5) $$\begin{array}{c} \left(t_{2}-t_{1}\right)=d+n_{ms},\;\left(t_{4}-t_{3}\right)=d+n_{sm} \end{array}$$

Substituting these into Equation (4), the delay terms cancel out, isolating the ideal quantization noise component $N_{ideal}$, as follows:

(6) $$\begin{array}{c} N_{ideal}=\frac{1}{2}\left(n_{ms}-n_{sm}\right) \end{array}$$

Statistically, the probability density function (PDF) of $N_{ideal}$ is the convolution of two uniform distributions, yielding a Triangular Distribution Λ(z) defined on $\left[-T_{clk},T_{clk}\right]$.

However, real-world implementations exhibit Gaussian-like error distributions due to noise superposition. The observed error $N_{obs}$ consists of the quantization error $N_{ideal}$ and an aggregate physical noise term $N_{phy}$ as follows:

(7) $$\begin{array}{c} N_{obs}=N_{ideal}+N_{phy}=N_{ideal}+\sum_{k=1}^{K}\xi_{k} \end{array}$$

Here, $\xi_{k}$ represents independent physical impairments (e.g., thermal noise, PHY transceiver latency asymmetry, PLL jitter) with variance $\sigma_{k}^{2}$. By the Central Limit Theorem, their sum converges to a normal distribution for a sufficiently large number of noise sources *K* as follows:

(8) $$\begin{array}{c} N_{phy}=\sum_{k=1}^{K}\xi_{k}\overset{d}{\to }N\left(0,\sigma_{phy}^{2}\right),\;\mathrm{where}\;\sigma_{phy}^{2}=\sum_{k=1}^{K}\sigma_{k}^{2} \end{array}$$

Consequently, the observed error PDF $P_{obs}\left(z\right)$ is the convolution of the triangular distribution Λ(z) and the Gaussian density ψ(z) as follows:

(9) $$\begin{array}{c} P_{obs}\left(z\right)=\left(\Lambda *\psi \right)\left(z\right)=\int_{-\infty }^{\infty }\Lambda \left(z-\tau \right)\frac{1}{\sqrt{2\pi }\sigma_{phy}}e^{-\frac{\tau^{2}}{2\sigma_{phy}^{2}}}d\tau \end{array}$$

When $\sigma_{phy}$ is comparable to $T_{clk}$, this convolution smooths the triangular shape into a quasi-Gaussian profile. Note that while modeled stochastically, the frequency difference induces a beat frequency effect, creating a deterministic sawtooth pattern in quantization error, which motivates the need for predictive compensation.

### 3.3. Optimization Problem

The objective is to minimize time errors using software-based frequency modulation under measurement noise. At step *k*, the system obtains a noisy measurement $y_{k}$, which corresponds to the PTP offset corrupted by the noise realization $N_{obs}\left(k\right)$ as follows:

(10) $$\begin{array}{c} y_{k}=x\left(t_{k}\right)+N_{obs}\left(k\right) \end{array}$$

Standard PTP servos employ frequency adjustment rather than instantaneous phase resets to maintain clock stability. A compensation rate $u_{k}$ is applied over the interval $\left[t_{k},t_{k+1}\right)$. The true time error x(t) evolves according to the following:

(11) $$\begin{array}{c} x\left(t_{k}+\tau \right)=x\left(t_{k}\right)+\int_{t_{k}}^{t_{k}+\tau }\alpha_{drift}\left(s\right)ds-u_{k}\cdot \tau \end{array}$$

Substituting the state estimate $x\left(t_{k}\right)=y_{k}-N_{obs}\left(k\right)$, the residual error E(t) at time τ is formulated as follows:

(12) $$\begin{array}{c} E\left(t_{k}+\tau \right)=y_{k}-N_{obs}\left(k\right)+\int_{t_{k}}^{t_{k}+\tau }\alpha_{drift}\left(s\right)ds-u_{k}\cdot \tau \end{array}$$

This residual error comprises the measured offset $y_{k}$, the unobservable quantization noise $-N_{obs}\left(k\right)$, stochastic drift accumulation, and the deterministic compensation $-u_{k}\tau$.

To minimize the residual error at a specific horizon $\tau_{target}$, setting $E(t_{k}+\tau_{target})=0$ yields the theoretically optimal compensation rate $u_{k}^{*}$ as follows:

(13) $$\begin{array}{c} u_{k}^{*}=\frac{y_{k}}{\tau_{target}}+\frac{1}{\tau_{target}}\left(\int_{t_{k}}^{t_{k}+\tau_{target}}\alpha_{drift}\left(s\right)ds-N_{obs}\left(k\right)\right) \end{array}$$

In Equation (13), the first term is the reactive compensation (standard PTP), while the second term represents predictive compensation for future drift and current noise. Since $\alpha_{drift}$ and $N_{obs}\left(k\right)$ are latent variables, $u_{k}^{*}$ cannot be computed directly.

Consequently, we formulate the optimization problem to find a policy π—mapping historical observations to actions $u_{k}$—that minimizes the expected mean squared residual error over an infinite horizon as follows:

(14) $$\begin{array}{c} \min_{\pi }J\left(\pi \right)=\lim_{K\to \infty }\frac{1}{K}\sum_{k=0}^{K-1}E\left[{\left|E(t_{k}+\tau_{target})\right|}^{2}\right] \end{array}$$

By minimizing the expected squared residual error of E, the policy π implicitly estimates the unobservable dynamics to drive $u_{k}$ towards the optimum $u_{k}^{*}$.

## 4. Deep Reinforcement Learning-Enhanced Precise Time Synchronization

### 4.1. DRL-Based Control Framework

For the optimization objective derived in Equation (14), the timestamping quantization noise magnitude is comparable to, or slightly exceeds, the short-term frequency drift accumulation. In this regime, conventional linear controllers and supervised learning methods face fundamental theoretical limitations. It is critical to distinguish between the statistical and temporal properties of the noise as follows: while Section 3 derived the triangular distribution based on the assumption of spatial independence per transaction, the errors exhibit strong temporal autocorrelation in continuous operation due to the beat frequency effect. PID controllers fail to exploit this structure, suffering from a bandwidth trade-off where they treat this correlated quantization pattern as valid signal to be tracked or high-frequency noise to be suppressed, unable to simultaneously achieve both. Similarly, Kalman Filters prove suboptimal due to their reliance on Gaussian white noise assumptions. They treat the deterministic sawtooth pattern (hidden within the triangular statistics) as random uncertainty, thereby averaging out the predictable quantization structure rather than compensating for it. Furthermore, supervised neural networks are rendered ineffective in this direct connection scenario. Since the aggregate magnitude of timestamping and path noise is approximately equal to the true time error, the signal-to-noise ratio is prohibitively low. Static supervised models cannot decouple the valid drift signal from the composite noise without the temporal context of interaction, making gradient optimization intractable. Consequently, the solution requires a Reinforcement Learning algorithm that progressively extracts this hidden state information through active interaction with the environment.

To address this tractability challenge, we propose a Deep Reinforcement Learning (DRL) framework formulated as a Partially Observable Markov Decision Process. The synchronization controller functions as the agent, actively interacting with the environment. Unlike analytical methods that require explicit noise modeling, the DRL agent approximates the inverse function of the error evolution dynamics. By learning a policy $\pi_{\theta }\left(a_{t}|s_{t}\right)$ via an LSTM-based encoder, the agent captures the temporal dependencies of the sawtooth wave, generating frequency compensation rates to minimize the expected residual error $E[|E{|}^{2}]$. This effectively compensates for both the stochastic drift trend and the predictable component of the quantization noise that linear filters miss.

### 4.2. State Space

We assume the ambient temperature is time-invariant. The state $s_{t}$ at time step *t* is constructed using historical sequences of measured clock offsets and path delays. To capture the temporal dependencies inherent in the drift process, the state is defined over a history window *w* as follows:

(15) $$\begin{array}{c} s_{t}=\left[o_{t-w}^{t},d_{t-w}^{t}\right] \end{array}$$

where $o_{t-w}^{t}$ denotes the sequence of measured offsets $\left\{o_{t-w},\ldots ,o_{t}\right\}$ and $d_{t-w}^{t}$ represents the sequence of path delays over the window *w*.

### 4.3. Action Space

We use $a_{t}$ to denote the action at time step *t*. At each synchronization interval, the agent selects an action $a_{t}$ to adjust the current compensation rate based on the current observed state $s_{t}$. Given the pre-measured time-error range of ±15 ns and considering that the offset error is close to the time error (quantized to integer ns on the NIC), we define the action space as follows:

(16) $$a_{t}\in \left\{0,\begin{array}{c} \pm 1 \end{array},\pm 2,\pm 4,\pm 8\right\}\;\begin{array}{c} \left(\mathrm{Unit}:\;\mathrm{ns}/s\right) \end{array}$$

### 4.4. Reward Function

**Figure 2:**
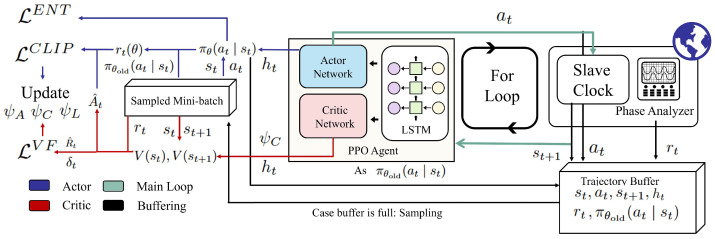
Workflow of the proposed PPO-LSTM scheme.

The reward function serves as the numerical surrogate for the optimization objective J(π) defined in Equation (14). During the training phase, the ground truth time error is obtained directly from the external high-precision phase analyzer, as illustrated in Figure 2.

We formulate the reward $r_{t}$ using a Gaussian kernel to encourage high-precision convergence, augmented with a penalty term to ensure training stability as follows:

(17) $$r_{t}=\exp\left(-\frac{\begin{array}{c} {\mathrm{TE}}_{t}^{2} \end{array}}{2\sigma_{target}^{2}}\right)-\beta_{Penalty}\cdot I\begin{array}{c} \left(|{\mathrm{TE}}_{t}|>\delta \right) \end{array}$$

In Equation (17), ${\mathrm{TE}}_{t}$ represents the residual true time errors measured at the end of the synchronization interval. The parameter $\sigma_{target}$ controls the sensitivity of the reward to small errors, effectively shaping the optimization landscape to prioritize nanosecond-level precision. The second term introduces a stability constraint, where I(·) is an indicator function that applies a penalty weighted by $\beta_{Penalty}$ if the error magnitude exceeds a safety threshold δ. This mechanism prevents the exploration policy from drifting into unstable regimes and facilitates the agent’s recovery during the early stages of learning.

### 4.5. Proximal Policy Optimization with Long Short-Term Memory Layers

To solve the optimization problem, we adopt a Deep Reinforcement Learning framework based on the Actor–Critic architecture, integrated with Long Short-Term Memory (LSTM) networks to capture the temporal dependencies in clock drift sequences. The architecture consists of a shared LSTM feature extractor parameterized by $\psi_{L}$, followed by the following two distinct branches: the Actor network and the Critic network. The Actor network defines a stochastic policy $\pi (a_{t}|s_{t},h_{t};\psi_{A})$ parameterized by $\psi_{A}$, which outputs the action probability distribution based on the current state $s_{t}$ and the LSTM hidden state $h_{t}$. Conversely, the Critic network estimates the value function $V(s_{t},h_{t};\psi_{C})$ parameterized by $\psi_{C}$, evaluating the expected return of the current state.

To ensure robust convergence and training stability, we utilize the Proximal Policy Optimization (PPO) algorithm [20] to train the proposed architecture. Unlike standard Advantage Actor–Critic (A2C) methods, PPO introduces a clipped surrogate objective function that constrains the magnitude of policy updates, preventing destructive large steps that could destabilize the clock servo. Furthermore, the algorithm employs Generalized Advantage Estimation (GAE) to significantly reduce the variance of advantage estimates. The complete workflow of the proposed PPO-LSTM scheme is illustrated in Figure 2. The advantage function is computed as follows:

(18) $$A^{GAE}\left(s_{t}\right)=\sum_{l=0}^{T-t-1}{\left(\gamma \lambda \right)}^{l}\delta_{t+l}$$

where $\delta_{t}=r_{t}+\gamma V\left(s_{t+1};\psi_{C}\right)-V\left(s_{t};\psi_{C}\right)$ is the temporal difference (TD) error, and λ is the GAE parameter. This multi-step advantage estimation provides a better bias-variance trade-off than single-step TD errors.

The training process employs an on-policy rollout buffer with mini-batch updates. In every episode of *T* time slots, the agent collects trajectories in buffer *B* containing $\left(s_{t},a_{t},r_{t},\pi_{t},h_{t},s_{t+1}\right)$ tuples, and it then computes discounted returns ${\hat{R}}_{t}=\sum_{k=0}^{T-t-1}\gamma^{k}r_{t+k}$. The optimizer performs *K* epochs of optimization over shuffled mini-batches using the following loss functions:

(19) $$\begin{array}{cc} L^{CLIP} & =E_{t}\left[\min(\rho_{t}A_{t},\mathrm{clip}\left(\rho_{t},1\pm \epsilon \right)A_{t})\right] \end{array}$$

(20) $$\begin{array}{cc} L^{VF} & =\frac{1}{2}E_{t}\left[{\left(V\left(s_{t};\psi_{C}\right)-{\hat{R}}_{t}\right)}^{2}\right] \end{array}$$

(21) $$\begin{array}{cc} L^{ENT} & =E_{t}\left[-\pi \left(s_{t};\psi_{A}\right)\log\pi \left(s_{t};\psi_{A}\right)\right] \end{array}$$

where $\rho_{t}=\frac{\pi_{\mathrm{new}}\left(a_{t}|s_{t}\right)}{\pi_{\mathrm{old}}\left(a_{t}|s_{t}\right)}$ is the probability ratio, and ϵ is the clipping threshold. This clipped objective prevents destructive large policy updates while maintaining training stability.

The final parameter updates combine these components with learning rates $\alpha_{A},\alpha_{C}$ and entropy weight β as follows:

(22) $$\begin{array}{cc} \psi_{A} & \leftarrow \psi_{A}+\alpha_{A}\left(\nabla_{\psi_{A}}L^{CLIP}+\beta \nabla_{\psi_{A}}L^{ENT}\right)\; \end{array}$$

(23) $$\begin{array}{cc} \psi_{C} & \leftarrow \psi_{C}-\alpha_{C}\nabla_{\psi_{C}}L^{VF}\; \end{array}$$

(24) $$\begin{array}{cc} \psi_{L} & \leftarrow \psi_{L}-\alpha_{C}\nabla_{\psi_{L}}L^{VF}+\alpha_{A}\left(\nabla_{\psi_{L}}L^{CLIP}+\beta \nabla_{\psi_{L}}L^{ENT}\right) \end{array}$$

We summarize the PPO-based scheme to solve the optimization problem specified by Equation (14) in Algorithm 1. Since we aim to minimize the expectations of time errors over time, we solve the optimization problem by selecting an action from our predicted actions.
**Algorithm 1** Proximal Policy Optimization (PPO) with LSTM for real-time control1:**Initialize**: Actor, Critic network and LSTM parameters $\psi_{A},\psi_{C},\psi_{L}$, hidden state *h*, buffer *B*2:**for** episode =1,2,… **do**3:       Clear hidden state: $h_{t}\leftarrow 0$4:       **for** t=0,…,T−1 **do**5:              Observe state $s_{t}$, obtain $h_{t}$ by shared LSTM layer.6:              Actor network calculates the Compensation probability distribution for state $s_{t}$ by using the policy function $\pi (s_{t},h_{t};\psi_{A})$, denoted as $\pi_{t}$.7:              The critic network evaluates state $s_{t}$ by using the value function $V(s_{t},h_{t};\psi_{C})$, denoted as $V_{t}$.8:              The agent samples $a_{t}∼\pi_{t}$ and executes $a_{t}$ in environment, then gets reward $r_{t}$ by Equation (17) and gets state $s_{t+1}$.9:              Store $\left(s_{t},a_{t},r_{t},\pi_{old},h_{t},s_{t+1}\right)$ in *B*.10:       **end for**11:       Compute returns ${\hat{R}}_{t}=\sum_{k=0}^{T-t-1}\gamma^{k}r_{t+k}$.12:       Compute generalized Advantage Estimation $A_{t}$ by Equation (18).13:       **for** k=1 **to** *K* **do**14:              Sample a mini-batch M⊂B.15:              Recompute $\pi_{\mathrm{new}}\left(a_{t}|s_{t}\right)$ for all transitions.16:              Compute ratios $\rho_{t}=\frac{\pi_{\mathrm{new}}\left(a_{t}|s_{t}\right)}{\pi_{\mathrm{old}}\left(a_{t}|s_{t}\right)}$.17:              Compute policy loss, value loss and entropy loss by Equations (19)–(21).18:              Update Network parameters using Equations (22)–(24).19:       **end for**20:**end for**

## 5. Experiment and Evaluation

### 5.1. Environment Setup

To measure the distribution of timestamping errors, we employed a high-precision, GNSS-locked phase error measuring instrument. A loopback configuration was established between two ports of the instrument to capture both bidirectional and master-to-slave time errors.The time synchronization cycle was set to a high frequency (1/128 s). At this rapid synchronization rate, the cumulative effect of frequency drift is negligible. Therefore, the measured unidirectional and bidirectional time errors are considered free of drift components and can be directly attributed to the current timestamping inaccuracies.

To validate the performance of the proposed time synchronization scheme, we use a NIC serving as the slave clock and the high-stability clock equipped with a rubidium oscillator locked to the GNSS as the master clock. Both the master and slave clocks support hardware timestamping. The master and slave clocks are interconnected via a 1.2-m Ethernet cable and the time synchronization cycle is set to 1 s. The slave clock hosts an agent that monitors the variations in offset and path delay during each synchronization cycle. Following each observation phase, the agent generates a compensation value and injects it into the protocol stack. A phase error measuring instrument is deployed between the master and slave clocks to quantify the true time discrepancy. This measured error is fed back in real time to the agent at the slave node, where it serves as the basis for reward computation in the synchronization optimization process. For comparative analysis, we also implemented a standard PID controller and a simple supervised LSTM predictor as baselines under identical conditions. The algorithm parameters are given in Table 1.

### 5.2. Probability Distribution of Hardware Timestamping Error

**Figure 3:**
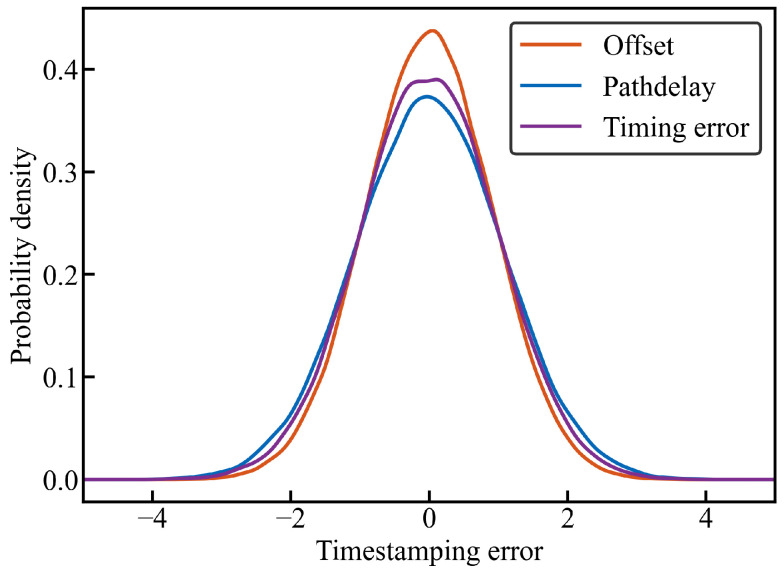
PDF of offset, path delay, and time errors.

In the experimental setup described in the previous subsection, we conducted a two-layer PTP synchronization test over a duration of $10^{4}$ s. After subtracting the mean values from the measured offset, path delay, and time errors, we fitted distribution curves to the data. The experimental results (Figure 3) demonstrate that the offset, time errors, and path delay all follow a normal distribution with a variance of approximately 1 ns, primarily distributed within ±2 ns. These findings are consistent with the hardware specifications. The Gaussian distribution differs from the idealized triangular distribution of pure quantization noise. This discrepancy validates our theoretical analysis in Section 3.2 as follows: the observed error is a composite of quantization noise and aggregate physical noise (e.g., thermal noise and PLL jitter). According to the Central Limit Theorem, the superposition of these independent physical noise sources smooths the triangular quantization component, resulting in the observed quasi-Gaussian distribution.

### 5.3. Result and Evaluation

**Figure 4:**
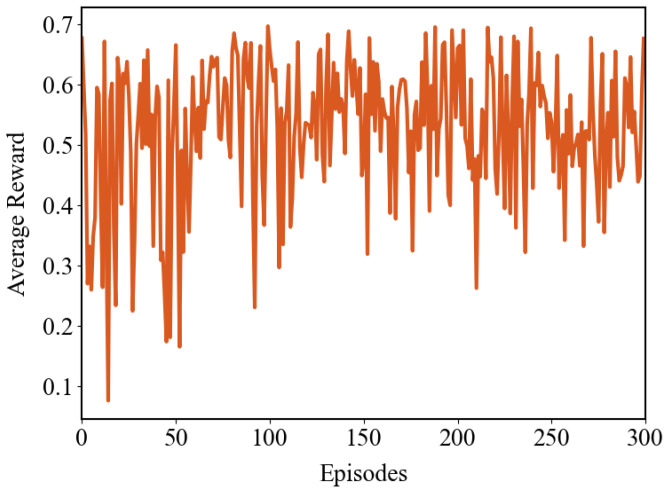
Trend of mean reward convergence during the training phase.

After 2 h of training, the time errors show significant improvement. As illustrated in Figure 4, the variation of rewards during the first 300 training episodes is presented, where each episode’s reward is calculated by averaging the rewards over 16 synchronization cycles. Overall, the reward exhibits an upward trend and gradually stabilizes, reflecting the reduction in error.

**Figure 5:**
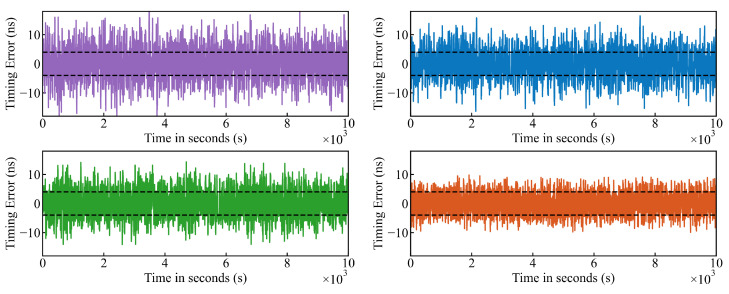
Comparison of time error sequences using Baseline (purple), PID Controller (blue), LSTM Compensation (green), and DRL Controller (orange) methods.

Figure 5 demonstrates the time errors trajectories across the following four different scenarios: Baseline (Open-Loop), PID Controller, LSTM Compensation, and the proposed DRL Controller. It can be observed that the error distribution of the DRL method is confined to a smaller range, with the system’s Maximum Time Interval Error (MTIE) significantly reduced from ±15 ns to ±10 ns. While the PID and LSTM methods show improvement over the baseline, the DRL controller exhibits the tightest control over the error amplitude. This improvement is attributed to the model’s ability to effectively mitigate the impact of timestamping errors when transitioning from maximum to minimum values.

**Figure 6:**
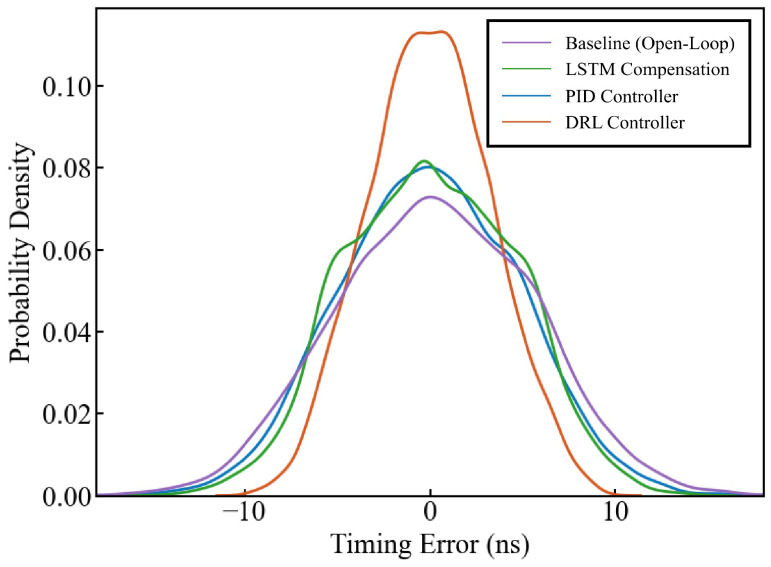
Comparison of the PDF of time errors using the Baseline, PID, LSTM, and DRL methods.

To better illustrate the algorithm’s effectiveness, Figure 6 presents the probability distribution of time errors before and after compensation. The Root Mean Square (RMS) error and probability distribution for critical time errors ranges are detailed in Table 2. The proposed DRL controller achieves the lowest RMS of 3.29 ns, outperforming the PID (4.77 ns) and LSTM (4.50 ns) approaches. Notably, the probability of time errors falling within ±1 ns, ±2 ns, ±3 ns, and ±4 ns for the DRL controller is significantly higher than other methods, reaching 76.13% within ±4 ns, demonstrating a clear optimization effect.

**Figure 7:**
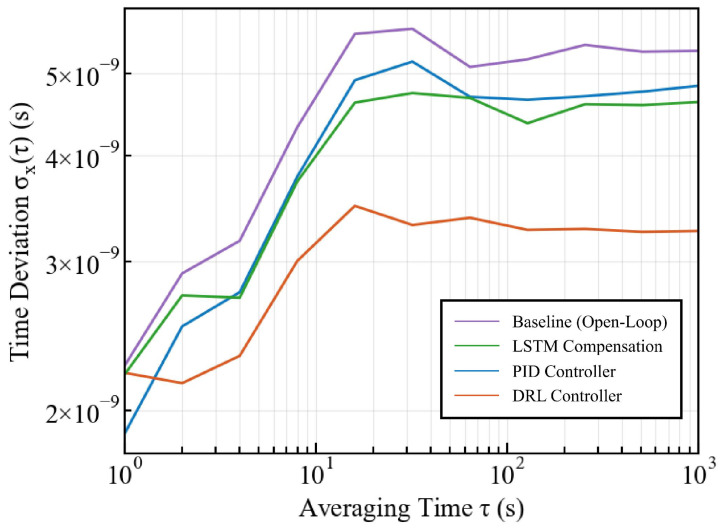
Comparison of TDEV stability analysis using the Baseline, PID, LSTM, and DRL methods.

Furthermore, we analyze the clock stability using Time Deviation (TDEV) as shown in Figure 7 and Table 3. While the PID controller exhibits a lower TDEV at the short-term integration time (τ=1), the DRL controller demonstrates superior long-term stability. As τ increases to 10, 100, and 1000, the DRL method consistently maintains the lowest TDEV values, indicating robust performance against long-term clock drift.

## 6. Conclusions

This paper analyzes the conditions for achieving nanosecond-level time synchronization for safety-critical functions in future intelligent transportation systems and conducts a detailed study on time errors. A time-error compensation method based on a deep reinforcement learning controller is proposed to compensate for clock offset, thereby eliminating frequency drift-induced time errors and timestamping errors, improving time synchronization accuracy. Experimental results demonstrate that this method reduces the RMS error to 3.29 ns compared to 4.77 ns with the PID and 4.50 ns with the LSTM methods. Additionally, it increases the probability distribution within ±4 ns by approximately 23% compared to the baseline, showing significant performance improvement and superior long-term stability as evidenced by TDEV analysis. Future work will extend this framework to multi-hop wireless scenarios where variable queuing delays and channel fading pose additional challenges to synchronization stability.

## Figures and Tables

**Table 1:** Hyperparameters and training configuration.

Category	Parameter	Value
Hardware & Env	Computing Platform	Ubuntu 20.04 LTS, 16-Core CPU
Reward Function	Penalty Weight ($\beta_{Penalty}$)	0.5
	Target Variance ($\sigma_{target}$)	4.0 ns
	Penalty Threshold (δ)	10 ns
PPO/A2C Algorithm	Actor Learning Rate ($\alpha_{A}$)	$3\times 10^{-4}$
	Critic Learning Rate ($\alpha_{C}$)	$1\times 10^{-3}$
	Discount Factor (γ)	0.99
	GAE Parameter (λ)	0.95
	Clipping Range (ϵ)	0.2
	Entropy Coefficient (β)	0.01
Model Architecture	Observation Window (*w*)	16 steps
	LSTM Hidden Units	128
	Mini-batch Size	64
Baseline Algorithms	PID Gains ($K_{p},K_{i},K_{d}$)	0.7, 0.3, 0.0
	Supervised LSTM Structure	2 Layers, 64 Units
	Supervised Learning Rate	$1\times 10^{-3}$

**Table 2:** Comparison of time error sequences using the Baseline, PID, LSTM, and DRL Methods.

	RMS	Cumulative Probability (%)			
Method	(ns)	±1 ns	±2 ns	±3 ns	±4 ns
Baseline (Open-Loop)	5.30	14.68	28.48	41.35	53.32
PID Controller	4.77	15.10	29.30	43.06	55.64
LSTM Compensation	4.50	16.07	30.86	44.97	58.13
DRL Controller	**3.29**	**22.16**	**43.60**	**61.59**	**76.13**

**Table 3:** TDEV stability analysis at specific integration times (τ).

	TDEV (ns)			
Method	τ=1	τ=10	τ=100	τ=1000
Baseline (Open-Loop)	2.2562	4.6904	5.1614	5.3205
PID Controller	**1.8751**	4.1109	4.6703	4.8380
LSTM Compensation	2.2032	3.9948	4.4773	4.6276
DRL Controller	2.2133	**3.1506**	**3.3056**	**3.2578**

## Data Availability

The original contributions presented in the study are included in the article; further inquiries can be directed to the corresponding author.
